# Widening of the electroactivity potential range by composite formation – capacitive properties of TiO_2_/BiVO_4_/PEDOT:PSS electrodes in contact with an aqueous electrolyte

**DOI:** 10.3762/bjnano.10.49

**Published:** 2019-02-15

**Authors:** Konrad Trzciński, Mariusz Szkoda, Andrzej P Nowak, Marcin Łapiński, Anna Lisowska-Oleksiak

**Affiliations:** 1 Faculty of Chemistry, Gdansk University of Technology, Narutowicza 11/12, 80-233 Gdansk, Poland; 2 Faculcty of Applied Physics and Mathematics, Gdansk University of Technology, Narutowicza 11/12, 80-233 Gdansk, Poland

**Keywords:** bismuth vanadate (BiVO_4_), electrochemical activity, PEDOT:PSS, supercapacitors, titania nanotubes

## Abstract

Composites based on the titania nanotubes were tested in aqueous electrolyte as a potential electrode material for energy storage devices. The nanotubular morphology of TiO_2_ was obtained by Ti anodization. TiO_2_ nanotubes were covered by a thin layer of bismuth vanadate using pulsed laser deposition. The formation of the TiO_2_/BiVO_4_ junction leads to enhancement of pseudocapacitance in the cathodic potential range. The third component, the conjugated polymer PEDOT:PSS, was electrodeposited from an electrolyte containing the monomer EDOT and NaPSS as a source of counter ions. Each stage of modification and deposition affected the overall capacitance and allowed for an expansion of the potential range of electroactivity. Multiple charge/discharge cycles were performed to characterize the electrochemical stability of the inorganic–organic hybrid electrode. Capacitance values higher than 10 mF·cm^−2^ were maintained even after 10000 galvanostatic cycles (*i*_c_ *= i*_a_ = 0.5 mA·cm^−2^).

## Introduction

Energy-storage technologies and sustainable energy production are currently important challenges. There are many ways for energy storage, among them, electrical, chemical and electrochemical storage technologies are of great interest [1–2]. Among the various energy storage devices, such as batteries [3] and supercapacitors [4], supercapacitors are the most promising candidates for storage because of fast charging/discharging processes, relatively simple structures, easy large-scale production and high power densities [5]. It is crucial to look for electrochemically stable electrode materials that exhibit high specific capacitance and can be rapidly and reversibly charged and discharged over a wide potential range. Both electrical double-layer capacitance and faradaic reactions can contribute to the final capacitance of an electrode. Many different materials have been tested as electrode materials for supercapacitors, such as metal oxides [6–8], carbon materials characterized by a developed surface area [9], graphene-based [10] and diamond-based materials [11], conductive polymers (CPs) and hybrid materials [12–13], and numerous types of composite materials [14–15]. For many years, conjugated polymers, also known as conductive polymers, e.g., poly(3,4-ethylenedioxythiophene) (PEDOT) have attracted great attention due to their high electrical capacitance even at very high charge/discharge rates [16]. The advantage of a conducting polymer over the other electrode materials, e.g., carbon-based electrodes, is that not only the surface but also the bulk of CPs gives an electrochemical response during the charge/discharge process. However, CPs suffer from a relatively narrow potential window of stability and electrochemical activity [17]. An extended potential range of electroactivity and an improvement of specific capacitance can be achieved, e.g., by the fabrication of organic–inorganic composites with TiO_2_ [18–19], organic–inorganic hybrids consisting of a conducting polymer and Prussian blue analogues [20], or composites with carbon nanomaterials [21]. Tuning of the electrochemical activity of supercapacitors can also be achieved via electrolyte modification. The addition of iodides to the electrolyte for carbon-based capacitors forms an additional carbon/electrolyte interface that contributes to the total capacity of the device [22–23] due to the faradaic reactions 3I_2_ + 2e^−^ → 2I_3_^−^ and I_3_^−^ + 2e^−^ →3I^−^ [24]. It is also known that the iodine/iodide couple is active on the surface of conjugated polymers [25].

In this study, we modified the surface layer of titania nanotubes and tested it as a potential electrode material for energy storage devices. The enhancement of pseudocapacitance, and the extension of the electroactivity range are the goals of this research. A nanotubular morphology of TiO_2_ was obtained by Ti plate anodization. The nanostructure of TiO_2_ provides a high specific surface area that is crucial for energy storage devices based on pseudocapacitance and electrochemical double-layer capacitance. TiO_2_ nanotubes were covered by a nanometric layer of bismuth vanadate obtained by pulsed laser deposition (PLD). It was recently reported that the TiO_2_/BiVO_4_ junction exhibits a synergistic effect towards photoelectrochemical water oxidation [26]. Further modification of the electrode material included hydrogenation. There are many ways to perform TiO_2_ hydrogenation [27–28], but in the present work, we utilized an electrochemical method. The last modification step of the TiO_2_/BiVO_4_ electrode the electrodeposition of PEDOT:PSS from an electrolyte containing the monomer EDOT and NaPSS as a source of counter ions. This procedure was expected to extend the electrochemical activity range and to improve the capacitance in comparison with pristine titania. The obtained electrode materials were electrochemically tested in an aqueous electrolyte. Multiple charge/discharge cycles (10000) were performed to test the electrochemical stability of the inorganic–organic hybrid and to determine its electrochemical capacitance.

## Experimental

### Apparatus

Raman spectra were obtained using a Renishaw InVia spectrometer with green laser excitation (514 nm) using a 50× LWD objective. The morphology of the samples was investigated by Schottky field-emission scanning electron microscopy (FEI Quanta FEG 250) with an ET secondary electron detector. The beam accelerating voltage was kept at 10 kV. Electrochemical measurements were recorded using the potentiostat–galvanostat system AutoLab PGStat204 under Nova 2.1 software control. The chemical composition measurements before and after the hydrogenation process were performed by using X-ray photoemission spectroscopy (XPS). The XPS measurements were performed using an Argus (Omicron NanoTechnology) X-ray photoelectron spectrometer. The photoelectrons were excited by a Mg Kα X-ray source. The X-ray anode was operated at 15 keV and 300 W. XPS measurements were performed at room temperature under ultrahigh-vacuum conditions, with pressures below 1.1 × 10^−8^ mbar. Data analysis was performed with the CASA XPS software package using Shirley background subtraction and a least-squares Gaussian–Lorentzian curve fitting algorithm. Obtained spectra were calibrated to give binding energy of 285.80 eV for C 1s [29–30].

### Chemicals

Titanium foil (Steam, 99.7%, *d* = 0.127 mm) was used as an electrode substrate. Chemicals of analytical grade, Bi(NO_3_)_3_·5H_2_O, NH_4_VO_3_, 3,4-ethylenedioxythiophene, poly(sodium 4-styrenesulfonate), NH_4_F were supplied by Sigma-Aldrich. K_2_SO_4_, H_2_SO_4_, ethylene glycol and H_3_PO_4_ were supplied by POCH. Triple distilled water was used for all electrochemical experiments.

### Electrochemical measurements

All electrochemical measurements were performed in 0.5 M K_2_SO_4_ aqueous electrolyte purged with argon. A three-electrode cell was used for cyclic voltammetry and galvanostatic charge–discharge cycles measurements. Platinum mesh acted as a counter electrode, and Ag/AgCl (0.1 M KCl) was used as the reference electrode. The geometric surface area of tested electrodes was equal to 0.5 cm^2^. Current densities used for charge/discharge tests were equal to *j*_k_ = *j*_a_ = 0.5 mA·cm^−2^. All values expressed by a surface containing unit are divided by the geometrical area of the electrode.

### Electrode preparation

#### Titania nanotubes

Titania nanotube synthesis was based on a two-stage anodization process in water/ethylene glycol (5%/95%) electrolyte containing 0.27 M NH_4_F and 1 M H_3_PO_4_ described previously [31]. The anodization process was performed in a two-electrode cell using platinum mesh as the cathode. The distance between the electrodes was about 2 cm. The anodization voltage was kept at 40 V for 2 h. The as-prepared electrodes were immersed for 12 h in 0.5 wt % aqueous solution of oxalic acid to remove the inhomogeneous layer of nanotubular TiO_2_. Then, the anodization procedure was repeated under the same conditions. Such a two-step procedure allows for a uniform nanotube layer to be obtained. Finally, samples were annealed at a temperature of 450 °C for 2 h (heating rate: 2 °C·min^−1^) in order to obtain crystalline TiO_2_. Electrodes were named as Ti/TiO_2_.

#### BiVO_4_

BiVO_4_ was synthesized via a solid-state chemical reaction using equimolar amounts of Bi(NO_3_)_3_·5H_2_O and NH_4_VO_3_. Ground powder was pressed into a pellet and annealed at 500 °C for 4 h. Then, the material was ground again and pressed in a hydraulic press (Specac Ltd) for about 60 s, with a load of about 35 MPa, into a pellet that acted as a PLD target. The procedure was developed and published previously [32]. PLD was used to deposit BiVO_4_ films on titania nanotubes (before annealing) or titanium foil. The bismuth vanadate films were deposited for 20 min and the PLD process was carried out using a Nd:YAG laser equipped with a fourth harmonic generation (FHG) module, generating 266 nm 6 ns pulses with 4 Hz repetition. Assuming that the obtained layer is flat and continuous, its thickness should be equal to about 20 nm [33]. Finally, electrodes were annealed at a temperature of 450 °C for 2 h (heating rate: 2 °C·min^−1^) in order to obtain crystalline phases of TiO_2_ and BiVO_4_. Samples were named as Ti/TiO_2_/BiVO_4_. The BiVO_4_ films were also deposited directly on titanium foil for comparison (Ti/BiVO_4_).

#### Hydrogenation process

Annealed titania nanotubes and titania nanotubes covered by BiVO_4_ were exposed to cathodic polarization in 1 M H_2_SO_4_ electrolyte. The potential of the working electrode during hydrogenation was equal to −1.5 V vs Ag/AgCl (0.1 M KCl) and the process lasted for 60 s. The hydrogenation process significantly affects TiO_2_ conductivity and specific capacitance [34]. After the hydrogenation process samples were named as Ti/TiO_2_:H/BiVO_4_:H.

#### PEDOT:PSS

**Figure 1:**
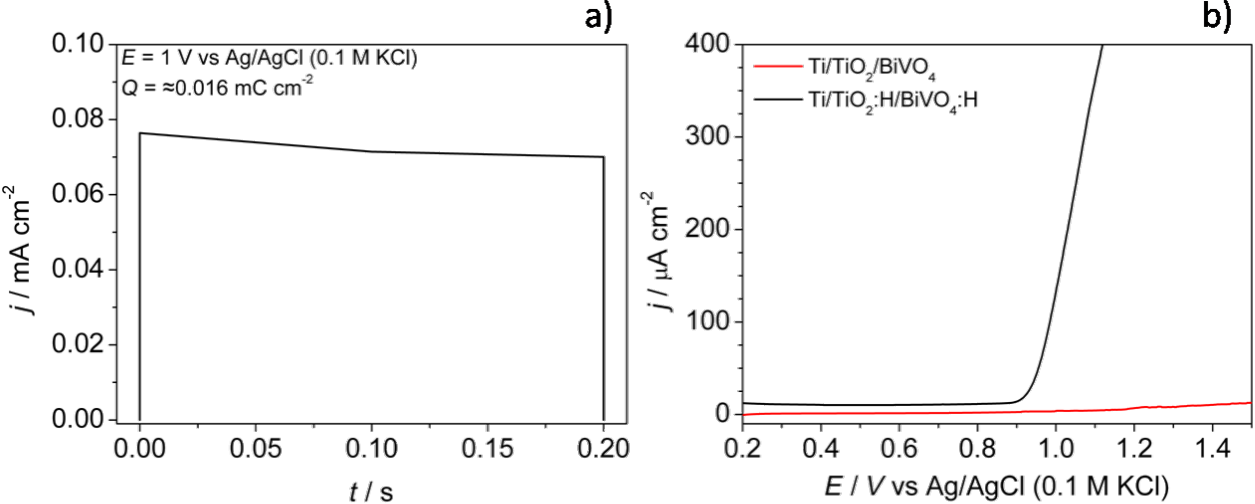
a) The chronoamperometry curve (single pulse at *E* = 1 V vs Ag/AgCl (0.1 M KCl)) recorded during PEDOT:PSS electropolymerization on a Ti/TiO_2_:H/BiVO_4_:H electrode and b) the linear sweep voltammograms recorded during polarization of Ti/TiO_2_/BiVO_4_ and Ti/TiO_2_:H/BiVO_4_:H electrodes in aqueous electrolyte containing monomer EDOT (1 mM) and NaPSS (0.1 M). Scan rate: 20 mV s^−1^, the electrode geometric surface area was equal to 0.5 cm^2^.

Poly(3,4-ethylenedioxythiophene):poly(styrenesulfonate) films were prepared by direct electropolymerization on a platinum foil or on Ti/TiO_2_:H/BiVO_4_:H electrodes from the aqueous electrolyte containing the monomer (1 mM EDOT) and poly(sodium 4-styrenesulfonate) (0.1 M NaPSS). The choice of PSS results from the assumption that large PSS ions built into the PEDOT matrix during the electrodepositon are not exchanged with anions originating from the electrolyte during electrooxidation/electroreduction cycles [35]. Moreover, the overpotential of EDOT oxidation is lower in NaPSS aqueous electrolyte (in comparison with, e.g., NaCl) [36]. Electrodepositon was performed using 400, short (0.2 s) potentiostatic pulses (*E* = 1 V vs Ag/AgCl (0.1 M KCl)). The charge consumed during polymerization was in the range from 0.01 to 0.1 mC·cm^−2^ per pulse, which is expected to deposit a conducting polymer film of 0.07 to 0.7 nm per pulse. An exemplary chronoamperometric curve (single pulse) is presented in Figure 1a. A Pt/PEDOT:PSS electrode was prepared for comparison.

Hydrogenation pretreatment was necessary to obtain PEDOT:PSS films directly on the Ti/TiO_2_/BiVO_4_ electrodes from the aqueous electrolyte. The comparison of linear sweep voltammograms recorded during EDOT oxidation on hydrogenated and nonhydrogenated TiO_2_/BiVO_4_ electrodes is shown in Figure 1b. The hydrogenation process significantly affects the EDOT oxidation potential and enables electropolymerization to occur directly on the modified titania nanotubes electrode surface. The electrodes after hydrogenation and PEDOT:PSS electrodepositon were simply named as Ti/TiO_2_/BiVO_4_/PEDOT:PSS. The Ti/TiO_2_/PEDOT:PSS electrode was prepared under the same (hydrogenation and PEDOT:PSS electrodeposition) conditions for comparison.

## Results and Discussion

### Structure and morphology

**Figure 2:**
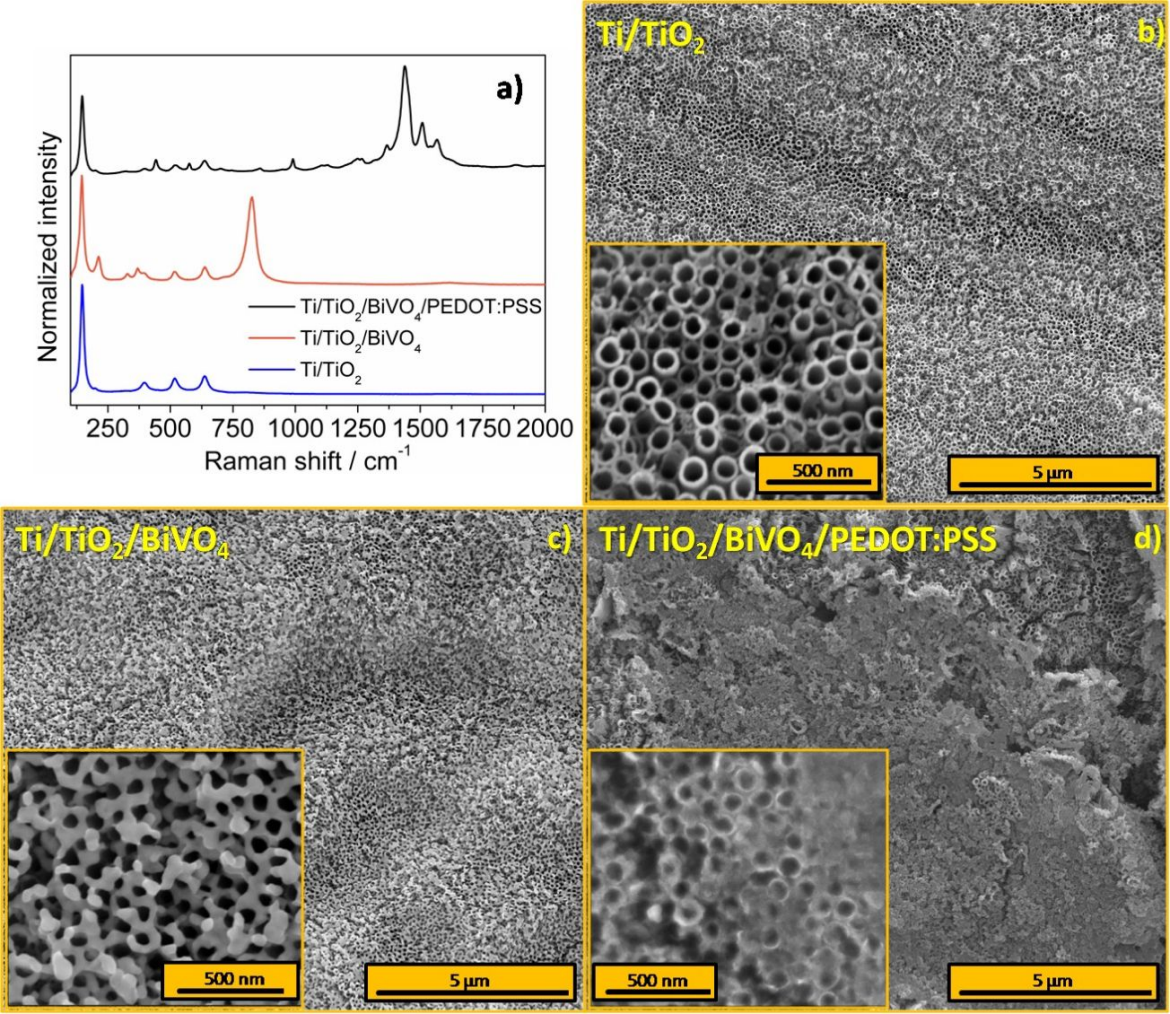
a) Raman spectra of Ti/TiO_2_ (blue), Ti/TiO_2_/BiVO_4_ (red), and Ti/TiO_2_/BiVO_4_/PEDOT:PSS (black). SEM micrographs of b) Ti/TiO_2_, c) Ti/TiO_2_/BiVO_4_, and d) Ti/TiO_2_/BiVO_4_/PEDOT:PSS. Insets in Figure 2b and 2c reprinted with permission from [26], copyright 2016 Elsevier.

The Raman spectra of annealed titania nanotubes and titania nanotubes with deposited BiVO_4_, as well as the composite Ti/TiO_2_/BiVO_4_/PEDOT:PSS, are presented in Figure 2a. There are five characteristic bands for the pure crystalline anatase phase for all samples. The bands were located at 144, 198, 395, 516 and 637 cm^−1^ and can be described as E_g(1)_, E_g(2)_, B_1g_, A_1g_, and E_g(3)_ active anatase modes, respectively [37]. The Raman spectrum of the electrode modified by bismuth vanadate also exhibited five bands at 212, 330, 368, 745 and 824 cm^−1^. These peak maxima are characteristic for the monoclinic scheelite structure of bismuth vanadate [38]. The band at the lowest Raman shift (ca. 212 cm^−1^) originates from the lattice mode. The bands at 330 and 368 cm^−1^, as well as 745 and 824 cm^−1^, can be attributed to the bending and stretching V–O vibrations, respectively [39–40]. Thus, the electrode preparation procedure leads to the formation of anatase (TiO_2_) and monoclinic scheelite (BiVO_4_) structures. Typical bands of PEDOT:PSS can be found in the Raman spectrum of the electrode after polymer electrodeposition. The main band (ca. 1438 cm^−1^) can be assigned to (C=C)–O vibrations in thiophene rings [41]. The symmetric C=C bands of the polymer chain are seen at 1508 and 1567 cm^−1^. The bands at ca. 1260 and 1367 cm^−1^ can be described as modes of carbon atoms with single bonds. The sharp peak at 989 cm^−1^ is assigned to the oxyethylene ring deformation band [41]. The position of the dominant band (1438 cm^−1^) and relatively high intensity of the peak at 1567 cm^−1^ suggest that the obtained polymer film is in its highly oxidized form [42]. Bands characteristic for BiVO_4_ were not detected for the samples covered by the polymer, probably due to the small amount of material and structure distortions. However, the presence of Bi and V was confirmed through XPS.

The morphology of the electrodes was studied by using scanning electron microscopy. The surface of the anodized titanium foil prepared according to the procedure described in the experimental part is presented in Figure 2b. Uniform coverage of titanium by titanium dioxide nanotubes was achieved. The average diameter and wall thickness of the nanotubes were estimated to be ca. 95 nm and ca. 20 nm, respectively (Figure 2c inset). The SEM micrograph of the Ti/TiO_2_/BiVO_4_ electrode material is presented in Figure 2d. PLD allows for a very homogenous deposition of bismuth vanadate. BiVO_4_ was mainly deposited on the upper edge of the titania nanotubes. The short time of BiVO_4_ deposition preserved the porous morphology of TiO_2_ nanotubes. It is advantageous that the nanoporous structure of the nanotubes is preserved and not fully filled with BiVO_4_. It ensures a large interface area between the electrode and the electrolyte that is crucial for electrodes for energy storage devices. TiO_2_ in the form of nanotubes is not only the template, but it also exhibits significant capacitive properties. Figure 2d shows the Ti/TiO_2_:H/BiVO_4_:H electrode after the PEDOT:PSS electrodeposition step. One may see the presence of structures originating from titania nanotubes. It evidences that the PEDOT:PSS film did not cover the whole electrode surface uniformly.

### XPS analysis

**Figure 3:**
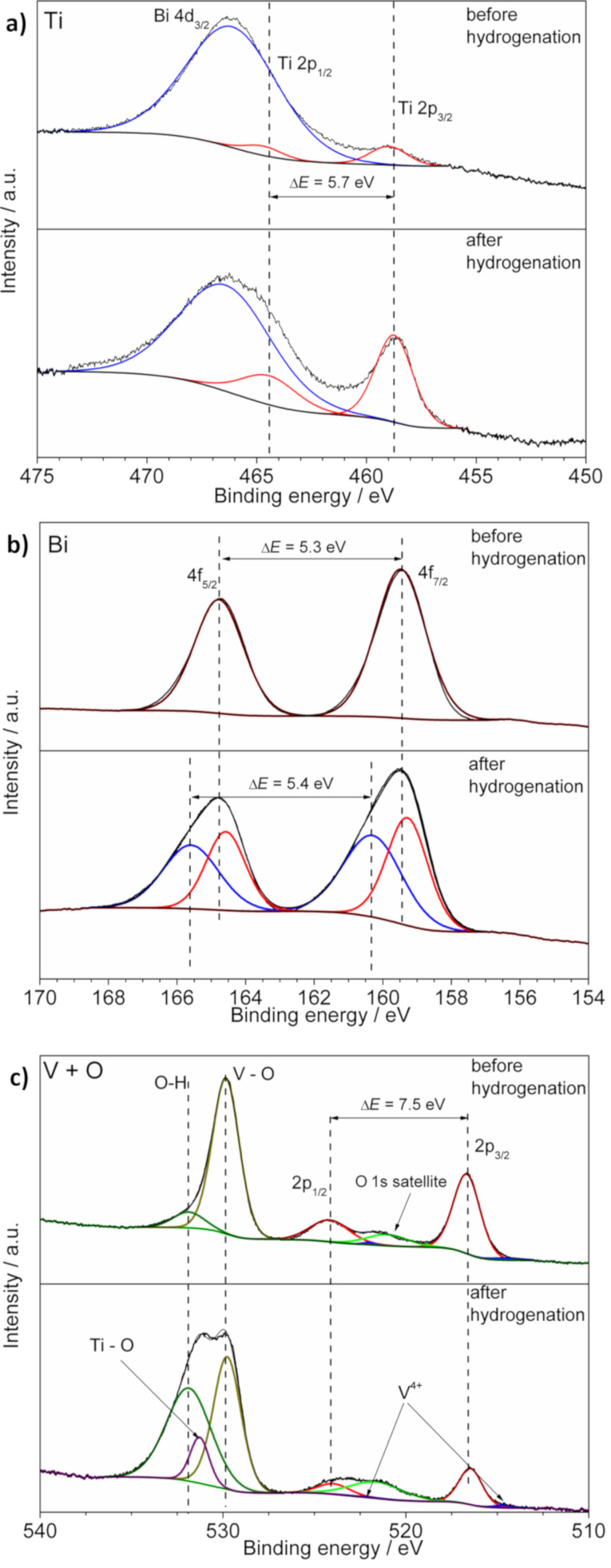
XPS spectra of a) Ti 2p region, b) Bi 4f region, and c) O 2p and V 2p regions for as-prepared and hydrogenated (Ti/TiO_2_/BiVO_4_ and Ti/TiO_2_:H/BiVO_4_:H) electrodes.

XPS analysis was performed in order to examine the influence of the hydrogenation process on TiO_2_ and BiVO_4_ electrode materials. The process of TiO_2_ hydrogenation was performed using different routes [43–44] and, as it was previously shown, it leads to the partial reduction of Ti^4+^ centers and the formation of Ti^3+^ [45]. On the other hand, here the XPS high-resolution Ti 2p spectrum of the Ti/TiO_2_:H/BiVO_4_:H sample shows that after the hydrogenation process titanium exists in the Ti^4+^ form (Figure 3a). One well-visible peak at 458.5 eV corresponds to Ti 2p_3/2_, while the Ti 2p_1/2_ peak is overlapping with the Bi 4d_3/2_ peak [46–47]. The weak Ti 2p_3/2_ peak visible in the spectrum of the as-prepared sample (Figure 3a) suggests the presence of only tetravalent titanium. Thus, hydrogenation of TiO_2_/BiVO_4_ did not clearly affect the bottom layer of TiO_2_. The XPS spectrum of the Bi 4f region of the sample before hydrogenation is characteristic for Bi^3+^ (Figue 3b). Deconvolution of the Bi 4f spectrum of the sample after the hydrogenation process exhibited an additional lower-intensity doublet at 160.3 eV (4f_7/2_) and 165.7 eV (4f_5/2_) besides the higher-intensity doublet at 159.3 eV (4f_7/2_) and 164.6 eV (4f_5/2_) (Figure 3b). That type of deconvoluted spectra might be attributed to the presence of higher oxidation states such as Bi^5+^ [48]. Moreover, the higher-intensity doublet is slightly shifted to lower energies, compared to the sample before hydrogenation, which could be caused by the presence of small amounts of reduced (in comparison with Bi^3+^) suboxides [49]. Such an effect was already observed for hydrogenated bismuth vanadate [50]. On the other hand, it could indicate that twin doublets might be associated with a more complicated chemical environment [51], and the electrode preparation process leads to Bi disproportionation, as it was already reported for other Bi-containing materials [52–53]. On the basis of V 2p spectra, one can see the good chemical stability of vanadium on the surface of BiVO_4_. XPS investigations showed that before and after hydrogenation vanadium is mostly V^5+^ with a very small contribution of V^4+^ (Figure 3c) [54]. The most significant influence of hydrogenation was observed in the O 1s region. Before the process, the spectrum consists of two peaks. The main peak at 529.8 eV can be attributed to oxygen in the BiVO_4_ crystal structure [55] and the smaller one is observed due to the presence of the surface OH groups [56]. The spectrum of the layer after hydrogenation is characterized by the much higher contribution of OH groups. There is also at least one additional peak that could be attributed to the titanium–oxygen bonds of titania [57].

### Electrochemical performance

**Figure 4:**
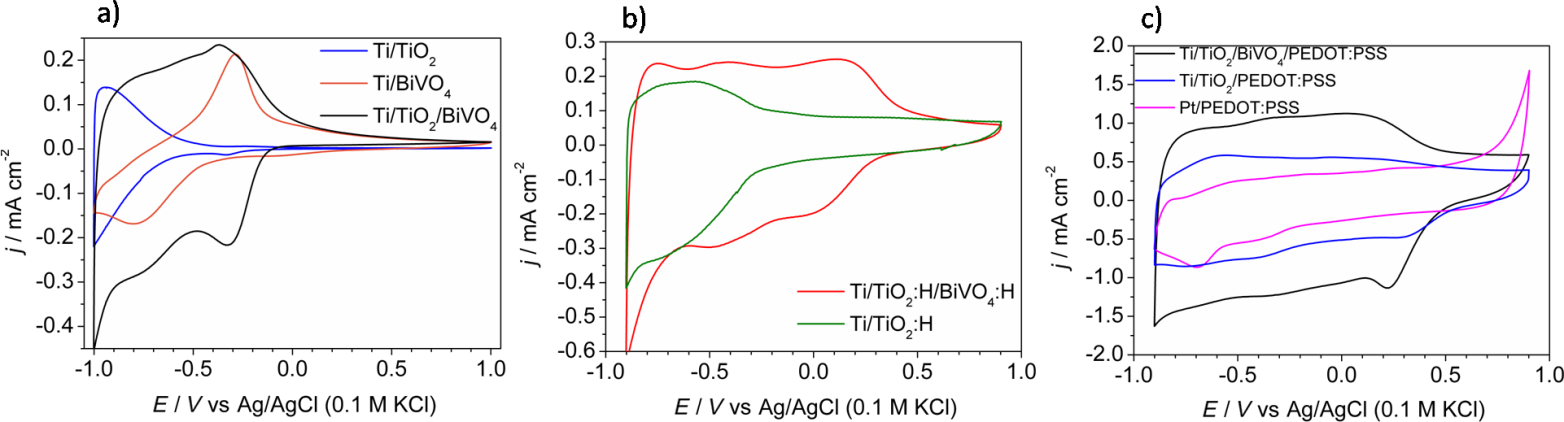
The cyclic voltammetry curves recorded in aqueous 0.5 M K_2_SO_4_ with scan rate 100 mV·s^−1^ of: a) Ti/TiO_2_, Ti/BiVO_4_, and Ti/TiO_2_/BiVO_4_, b) Ti/TiO_2_:H, Ti/TiO_2_:H/BiVO_4_:H, and c) Ti/TiO_2_/BiVO_4_/PEDOT:PSS, Ti/TiO_2_/PEDOT:PSS, and Pt/PEDOT:PSS.

Cyclic voltammetry was used for the electrochemical characterization of the electrode materials after each step of electrode preparation. The electrodes were tested in contact with an aqueous electrolyte. First, a comparison between Ti/TiO_2_, Ti/BiVO_4_, and Ti/TiO_2_/BiVO_4_ electrodes was made. The cyclic voltammetry (cv) curve of TiO_2_ nanotubes is presented in Figure 4a. The electrochemical activity of the TiO_2_ electrode material is clearly seen in the cathodic range of applied potential. It is related to the reduction/oxidation of titanium on the surface bonded to OH groups [58]. The reduction/oxidation activity is commonly described as H^+^ doping/dedoping with simultaneous reduction/oxidation of Ti^4+^/Ti^3+^ for TiO_2_ in the form of both nanotubes [59] and single crystals [60]. BiVO_4_ was deposited using the same PLD conditions, directly on titanium foil for comparison. The cv curve is characterized by cathodic and anodic maxima at −0.78 V and −0.29 V, respectively. Bismuth vanadate layers were already tested as an electrode material for energy storage devices and the mentioned maxima were attributed to the redox activity of the Bi/Bi^3+^ couple [61]. The combination of both materials, TiO_2_/BiVO_4_, was expected to increase the electric capacitance of the electrode in comparison with separate TiO_2_ and BiVO_4_. The cv curve of Ti/TiO_2_/BiVO_4_ presented in Figure 4a exhibited in the studied potential range (from −1 V to about −0.25 V) a rectangular shape, typical for electrodes in electrochemical capacitors. For further improvement of electrochemical activity and electrode response of Ti/TiO_2_ and Ti/TiO_2_/BiVO_4_ electrode materials, both substrates were modified through electrochemical hydrogenation. The phenomenon of hydrogenation of TiO_2_ and its influence on electrical, electrochemical and photoelectrochemical properties have been already reported [43,62]. Direct comparison of cv curves of Ti/TiO_2_ (Figure 4a) and Ti/TiO_2_:H (Figure 4b) showed that the hydrogenation process extremely extends the electroactivity range from Δ*E* = 0.9 V (from −1.0 V to −0.1 V) for Ti/TiO_2_ to Δ*E* = 1.9 V (from −1.0 V to 0.9 V) for Ti/TiO_2_:H. An extended range of potential where electrodes exhibit electrochemical activity yields a greater capacity. Moreover, due to the improvement of electrical properties, the Ti/TiO_2_:H/BiVO_4_:H electrode material could be further covered by conducting polymer prepared via electrodeposition. Beside many possible applications of conducting polymers like hole-transport material [63], electrochromic layers [64], electrochemical sensors [65] and gas sensors [66], a conducting polymer, particularly PEDOT, may be used as an electrode material for supercapacitors in both aqueous [67] and nonaqueous electrolytes [68].

In Figure 4c cv curves of Ti/TiO_2_/BiVO_4_/PEDOT:PSS, Ti/TiO_2_/PEDOT:PSS, and Pt/PEDOT:PSS electrode materials are compared. The range of electrochemical activity is wider for the composite electrodes in comparison with bare PEDOT:PSS on a platinum substrate due to PEDOT overoxidation [42], which starts to occur at a potential of *E* = 0.7 V only in the case of the Pt/PEDOT:PSS electrode. The Ti/TiO_2_/BiVO_4_/PEDOT:PSS and Ti/TiO_2_/PEDOT:PSS electrodes can be safely polarized from −0.9 V to 0.9 V without irreversible oxidation of the conducting polymer. Thus, an enormous value of Δ*E* = 1.8 V was achieved, which is a higher than the value of a PEDOT:PSS layer deposited on the Pt substrate (Δ*E* ≈ 1.6 V). The presence of BiVO_4_ in organic/inorganic composite electrode does not clearly affect the electroactivity potential range, because it overlaps with the electroactivity of PEDOT:PSS. However, current density (and capacitance) are higher for electrodes with added bismuth vanadate. Thus, one may conclude, that appropriate modification of the Ti/TiO_2_electrode significantly affects i) the range of electroactivity and ii) the stored charge capacity.

**Figure 5:**
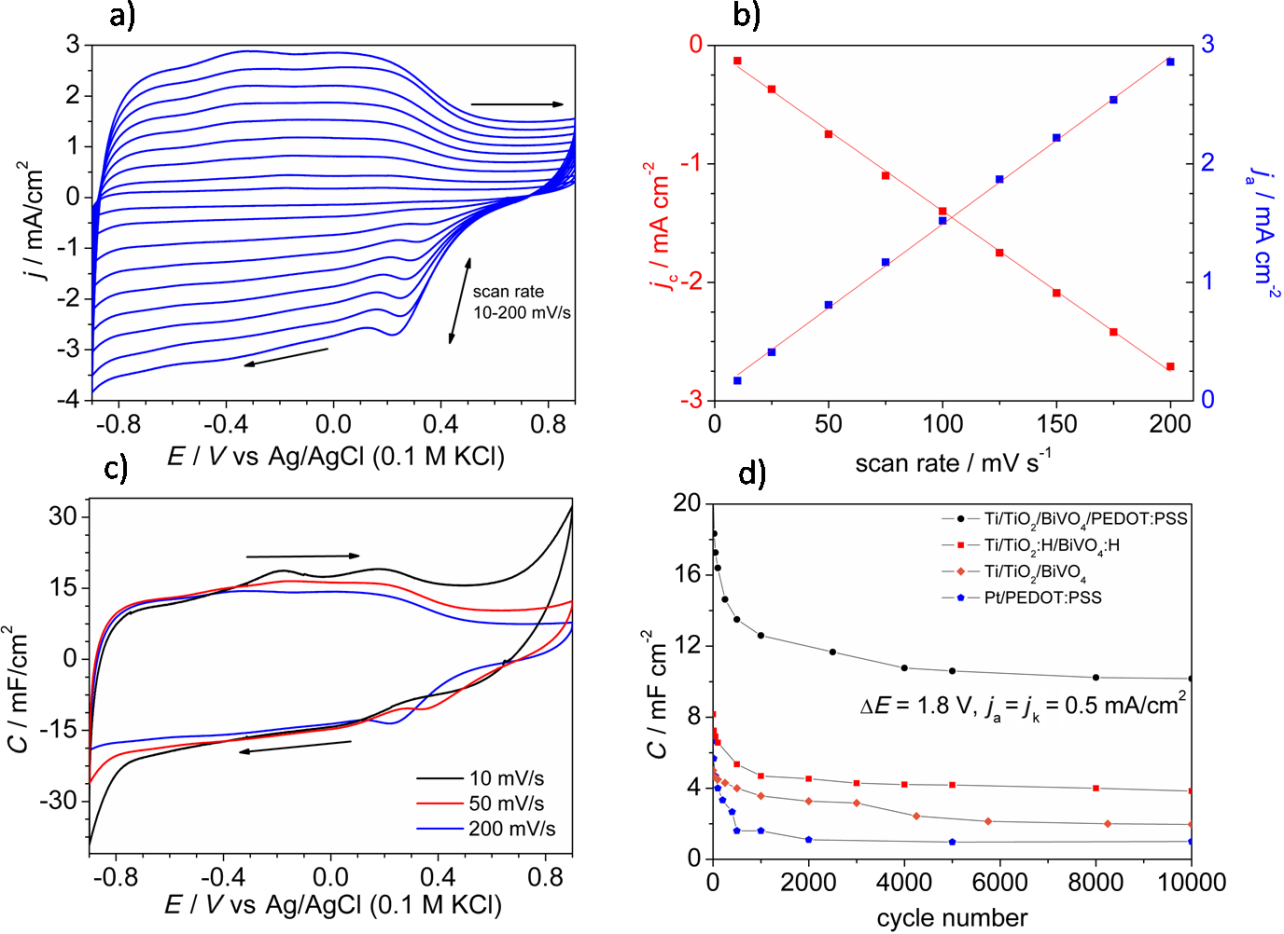
a) The cyclic voltammetry curves of Ti/TiO_2_/BiVO_4_/PEDOT:PSS recorded with various scan rates (10–200 mV·s^−1^). b) Dependence of current density at *E* = 0 V on the scan rate. c) Capacitance as a function of the potential calculated from cv curves. d) Capacitance as a function of the number of cycles resulting from chronopotentiometric cycles.

Further electrochemical measurements were performed for the Ti/TiO_2_/BiVO_4_/PEDOT:PSS electrode prepared via 400 potentiostatic pulses. The cv curves recorded at scan rates from 10 to 200 mV·s^−1^ are shown in Figure 5a. The rectangular shape of the cv curves is preserved for both low and high scan rates. Moreover, an almost linear relationship (*R*^2^ = 0.998) between scan rate and current density was obtained suggesting that prepared electrodes should not show a capacity drop as the current rate of charging/discharging increases, see Figure 5b. And indeed, the plots of capacitance as a function of the potential almost overlapped in the measured potential range for three different scan rates, as it is shown in Figure 5c.

The electrochemical stability of electrodes was characterized by multiple charge/discharge cycles performed for four types of electrodes: Pt/PEDOT:PSS, Ti/TiO_2_/BiVO_4_, Ti/TiO_2_:H/BiVO_4_:H, and Ti/TiO_2_/BiVO_4_/PEDOT:PSS. The obtained results are presented in Figure 5d. A drastic drop in capacitance during the first 500 cycles was observed for electrodes containing the conducting polymer film. In the case of the Pt/PEDOT:PSS electrode, the cohesion of the polymer layer to the platinum substrate after 500 cycles is very weak and the layer comes off easily. This was not observed for Ti/TiO_2_/BiVO_4_/PEDOT:PSS. The comparison of the electrochemical performance of Ti/TiO_2_/BiVO_4_ and Ti/TiO_2_:H/BiVO_4_:H is presented in Figure 5d. The capacitance at the beginning of the electrochemical test is about two times higher for the hydrogenated electrode, however, the capacitance retention, especially during first ca. 1000 cycles is significantly lower (63% and 72% for hydrogenated and non-hydrogenated samples, respectively). Despite the lower electrochemical stability, the capacitance even after 10000 cycles was still higher than that of the Ti/TiO_2_/BiVO_4_ electrode. The hydrogenation procedure affects the final capacitance of the Ti/TiO_2_/BiVO_4_/PEDOT:PSS electrode although the main aim of hydrogenation was to enable the polymer electrodeposition. Although the presence of PEDOT:PSS significantly enhanced the potential range of electrochemical activity and overall capacitance of the electrode, it affects negatively the electrode stability over multiple galvanostatic cycles. Nevertheless, despite a noticeable decrease of capacitance after the initial 1000 charge/discharge cycles (capacitance retention ca. 69% after 1000 cycles), the electrode named as Ti/TiO_2_/BiVO_4_/PEDOT:PSS exhibited a capacitance higher than 10 mF·cm^−2^ even after 10000 cycles. The capacity retention between the 1000th and the 10000th cycle was equal to 81%. The determined capacitance was over 10 times higher than for the Pt/PEDOT:PSS electrode and over 2.5 times higher in comparison with the Ti/TiO_2_/BiVO_4_ electrode material. Results are compared in Table 1. The enhancement of the measured capacitance of Ti/TiO_2_/BiVO_4_/PEDOT:PSS is mainly related to the presence of three different materials that contribute to the final capacitance. TiO_2_ and BiVO_4_ exhibit faradaic capacitance related to the Ti^4+^/Ti^3+^ [60] and Bi^3+^/Bi [69] redox couples, and PEDOT:PSS exhibits the pseudocapacitance coming from fast and reversible oxidation/reduction processes related to the π-conjugated polymer chains [70].

**Table 1:** The comparison of capacitance values for 1st, 1000th, and 10000th cycle. Capacity retention (CR) calculated between 1st and 1000th cycle, between 1st and 10000th cycle, and between 1000th and 10000th cycle.

Sample	*C*/mF·cm^−2^	*C*/mF·cm^−2^	*C*/mF·cm^−2^	CR	CR	CR
	1st cycle	1000th cycle	10000th cycle	1–1000	1–10000	1000–10000

Pt/PEDOT:PSS	5.6	1.6	1	28%	18%	62%
Ti/TiO_2_/BiVO_4_	5	3.6	2	72%	40%	55%
Ti/TiO_2_:H/BiVO_4_:H	8.2	5.2	3.8	63%	46%	73%
Ti/TiO_2_/BiVO_4_/PEDOT:PSS	18.3	12.6	10.2	69%	56%	81%

TiO_2_ in a form of nanotubes have been already tested as electrodes for supercapacitor working in a contact with an aqueous electrolyte. Pristine nanotubes exhibited capacitance equal to about 0.9 mF·cm^−2^ [71]. Commonly used hydrogenation procedure leads to the increase of capacitance to about 6 mF·cm^−2^ after few cycles for electrochemical high-temperature hydrogenation [72] and over 7 mF·cm^−2^ for titania nanotubes treated with hydrogen plasma [73]. A capacitance higher than 20 mF·cm^−2^ was obtained for electrochemically hydrogenated TiO_2_ nanotubes, but recorded only for very low current densities (0.05 mA·cm^−2^) [44]. The formation of a composite that consists of TiO_2_ and a conductive polymer for energy storage devices was reported. However, the measurements performed in aqueous electrolytes were recorded at much narrower potential range than presented in this work (not exceeding 1 V) [74–76]. Recently, it was reported that titania nanotubes/polyaniline composites in contact with aqueous electrolyte can be polarized in a range between −0.2 to 1.8 V vs SCE with very good capacitance retention [77], but the achieved areal capacitance (ca. 6 mF·cm^−2^) seems to be lower in comparison with Ti/TiO_2_/BiVO_4_/PEDOT:PSS presented here. Some authors show the results of capacitance per mass of the active electrode material. In the case of TiO_2_/polymer composites, the mass of the inorganic part is sometimes omitted [45,78]. However, such an approach could lead to a significant revaluation of capacitance. In the present work, the calculation of capacity by mass would require the estimation of the masses of TiO_2_ nanotubes, of the sputtered BiVO_4_ film and of the electrodeposited polymer. The direct weighing of nanostructures is very often difficult to perform due to the very small masses of sputtered material. In the case of bare titania nanotubes, the mass of the material could be determined on the basis of calculations taking into account dimensions of the tubes and the density of anatase. However, questionable is the use of anatase density in the case of such defected crystal structures. Also, the assumption that all nanotubes have the same size is a huge simplification. It could be also estimated by weight measurements of the electrode before and after chemical etching of the TiO_2_ nanotubes, e.g., using HF. However, selective detaching of nanotubes is difficult and there is a possibility to remove the TiO_2_ barrier and etch the metallic substrate as well. The determination of the mass of the nanometric films is also problematic in the case of BiVO_4_ deposited using PLD. Also, despite the electrochemical deposition of PEDOT:PSS, Faraday’s law cannot be simply utilized to calculate the mass of the polymer. The attempt of mass estimation could be performed on the basis of film thickness and density assuming a cuboid shape of the BiVO_4_ and PEDOT:PSS films. In the case of titania nanotubes prepared through anodization, one may estimate the gravimetric capacitance of the electrodes taking into account also the mass of the current collector (Ti foil). Depending on what is actually considered as the electroactive material, the gravimetric capacitance of the Ti/TiO_2_/BiVO_4_/PEDOT:PSS composite varies from 0.14 to 680 F·g^−1^, for the whole electrode with Ti foil and for PEDOT:PSS only, respectively. Thus, one may see that giving the capacitance value in farad per gram may lead to incorrect conclusions. Due to the difficulties to accurately determine the mass of the electroactive material and to avoid the uncertainty associated with the use of mass-specific capacitance, in the present work only the areal capacitance was discussed.

## Conclusion

This report is devoted to the design and investigation of a novel electrode material, Ti/TiO_2_/BiVO_4_/PEDOT:PSS, which is characterized by a high capacity during polarization in contact with an aqueous electrolyte. Here, we present the formation of a titania nanotube-based composite. The proposed procedure of anodization yields titania (anatase crystal structure) in the form of nanotubes. PLD was used for BiVO_4_ deposition on TiO_2_ with preservation of nanotubular morphology. An enhancement of electric-charge storage was clearly achieved at this stage of electrode modification. The Ti/TiO_2_/BiVO_4_ electrode material was further modified through a electrochemical hydrogenation process. This procedure extended the range of electroactivity and improved the storage ability, while simultaneously enabling the electrodeposition of PEDOT:PSS. Finally, Ti/TiO_2_/BiVO_4_/PEDOT:PSS electrodes were electrochemically tested over a wide range of potential (Δ*E* = 1.8 V). The electrode showed comparable capacity values over a wide range of polarization scan rates (10–200 mV·s^−1^). Multiple galvanostatic charge–discharge cycles lead to a 31% decrease in capacitance after the first 1000 cycles. Then, the capacitance stabilized and the value was maintained above 10 mF·cm^−2^ even after 10000 cycles with a capacity retention of 81%. Beside the capacitance enhancement, appropriate pretreatment of titania nanotubes allows the potential range of electroactivity to be tuned.
